# Shear Stress, Energy Losses, and Costs: A Resolved Dilemma of Pulsatile Cardiac Assist Devices

**DOI:** 10.1155/2014/651769

**Published:** 2014-01-08

**Authors:** Sayed Nour, Jia Liu, Gang Dai, Daniel Carbognani, Daya Yang, Guifu Wu, Qinmei Wang, Juan Carlos Chachques

**Affiliations:** ^1^Laboratory of Biosurgical Research (Alain Carpentier Foundation), Pompidou Hospital, University Paris Descartes, 56 Rue Leblanc, 75015 Paris, France; ^2^Division of Cardiology and the Key Laboratory on Assisted Circulation, Ministry of Health of China, The First Affiliated Hospital of Sun Yat-sen University, Guangzhou 510080, China

## Abstract

Cardiac assist devices (CAD) cause endothelial dysfunction with considerable morbidity. Employment of pulsatile CAD remains controversial due to inadequate perfusion curves and costs. Alternatively, we are proposing a new concept of pulsatile CAD based on a fundamental revision of the entire circulatory system in correspondence with the physiopathology and law of physics. It concerns a double lumen disposable tube device that could be adapted to conventional cardiopulmonary bypass (CPB) and/or CAD, for inducing a homogenous, downstream pulsatile perfusion mode with lower energy losses. In this study, the device's prototypes were tested in a simulated conventional pediatric CPB circuit for energy losses and as a left ventricular assist device (LVAD) in ischemic piglets model for endothelial shear stress (ESS) evaluations. In conclusion and according to the study results the pulsatile tube was successfully capable of transforming a conventional CPB and/or CAD steady flow into a pulsatile perfusion mode, with nearly physiologic pulse pressure and lower energy losses. This represents a cost-effective promising method with low mortality and morbidity, especially in fragile cardiac patients.

## 1. Introduction

Mechanical cardiac assist devices (CAD) disturb endothelial function and hemodynamics [1, 2]. These most probably occur as a consequence of steady-flow or inadequate pulsatile mode of perfusion that suppresses endothelial shear stress (ESS) [3].

Shear stress-mediated endothelial function controls vascular tone with plenty of mediators [4, 5] as well as vascular conditions through several processes like atherosclerosis and angiogenesis-apoptosis interdependency [6, 7].

In general, present CAD can be identified in two categories: *devices* that increase coronary blood flow during diastole in order to improve the oxygenation and thus the performance of the myocardium, that is, the intra-aortic balloon pump (IABP) and the enhanced external counterpulsation pump (EECP) [8, 9]; these devices must be synchronized with heartbeat and they are unsuitable in case of cardiac arrhythmia and *devices* that unload and bypass the heart pump either partially as achieved by left ventricular assist devices (LVAD), right ventricular assist devices (RVAD), and extracorporeal membrane oxygenation (ECMO) or completely like with biventricular assist devices, cardiopulmonary bypass (CPB) [10–12].

Conceptually, these devices are lumped models, designed for driving Newtonian compressible fluids inside closed pressurized hydraulic circuits implementing rigid tubes with fixed diameters [13, 14]. Meanwhile, in practices those devices are confronted with blood, which is a non-Newtonian fluid running inside flexible vessels with different geometries and controlled by vascular resistance. Thus, confrontation between these two opposite pressurized hydraulic circuits creates a vicious circle of momentum energy losses and endothelial dysfunction. Besides, installations conduits between cardiovascular tissues and CAD create dead space with important zones of energy losses [15].

Finally, permanent replacement of the heart with an artificial heart option is still a work in progress with short life expectancy, which limits its applications in specific categories, regarding body surface area (1.9 ± 0.22 m^2^), sex (95% men), and age (practically 0% children) [16, 17].

In a matter to overrule these side effects, different therapeutic strategies are currently applied, particularly with CPB such as *pharmacological supports* [18], *normothermia* [19], and *pulsatile* CPB [20]. However, those trials remain insufficient, as drugs promote further drawbacks [21]. Benefits of normothermia, that may be due to the stability of blood viscosity at 37°C [22], remain controversial [23], as the myocardium is already protected with cardioplegia. In addition, perfusion of organs microcirculation is protected with hemodilution according to the Fahraeus-Lindqvist effect [24]. Besides, current pulsatile CPB induces inadequate perfusion curves and momentum energy losses that necessitate specific materials and technology [25]. On the other hand, association of IABP with conventional CPB, as a cost-effective manner, creates turbulent zones from opposing flows and vascular complications [26–28].

These side effects may explain current popularity of conventional CPB [29], in addition to the *off-bypass technique*, which is still restricted to small groups of patients [30].

Therefore, an optimum cardiovascular perfusion device is still missing, because we cannot simulate properly a type-III passive ventricular pump with type-I (e.g., roller) or type-II (e.g., centrifugal) pumps [31, 32].

Alternatively, we are proposing a new concept of pulsatile CAD based on a fundamental revision of the entire circulatory system in correspondence with the physiopathology and law of physics.

**Figure 1:**
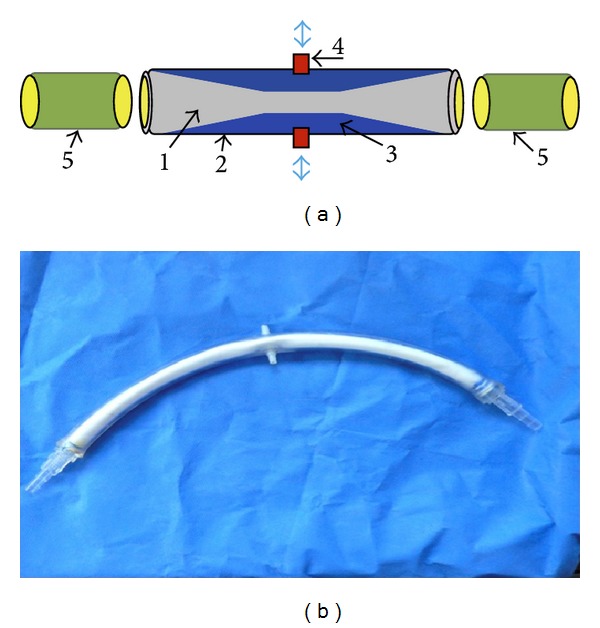
(a) Disposable pulsatile tube (pipe). According to patents descriptions (World Intellectual Property Organization: WO/2008/000110 and WO/2010/066899). 1 is flexible inner tube; 2 is rigid external tube; 3 is intermediate chamber; 4 is ports; 5 is connectors. (b) pulsatile tube prototype. External polyvinyl chloride (PVC) tube (1/2 inch); internal Polytetrafluoroethylene (PTFE) tube (12 mm), and connectors (1/16–1/4 inch).

As shown in (Figure 1(a)), it concerns a double lumen disposable tube device that could be adapted to conventional CPB and/or CAD, for inducing a homogenous, downstream pulsatile perfusion mode with lower energy losses.

In this study, device prototypes were tested in a simulated conventional pediatric CPB circuit for energy losses and as a LVAD in ischemic piglets model for ESS evaluations.

The goals of this study were to evaluate the feasibility of the proposed device to transform a steady-flow of conventional CAD into a pulsatile mode of perfusion with preservation of physiological ESS and minimum energy losses. This represents a cost-effective method with low morbidity and mortality.

## 2. Materials and Methods

*Device Prototype.* A double lumen tube (Figure 1(b)) was composed of (a) external polyvinyl chloride (PVC) (20 cm length, 1/2 inch diameter). (b) Internal polytetrafluoroethylene (PTFE) (18 cm length, 12 mm diameter) was reinforced with latex membrane (condom) to avoid any leakage through PTFE micropores. (c) 2 connectors (1/4 inch) were introduced at each end of the inner tube and securely sealed by external adhesive straps and rings in a wedged manner to the external PVC tube. A small animal ventilator (HX-300 TaiMeng Technologies Inc) was connected to the intermediate chamber through central holes at the external tube to be used as a pulsatile generator.

### 2.1. *In Vitro* Study

**Figure 2:**
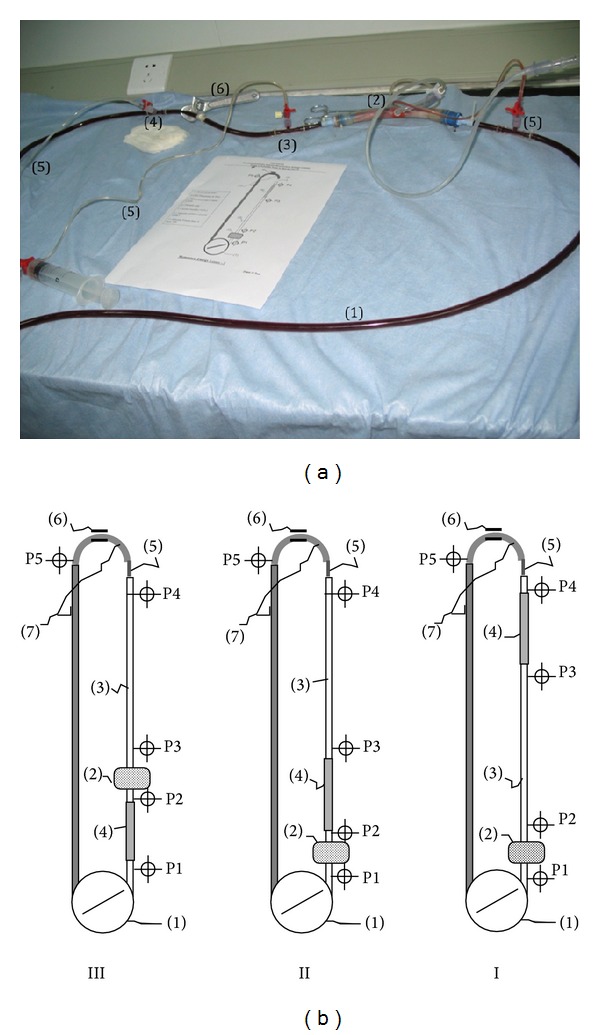
(a) Mock circulation: energy losses circuit (I). 1 is arterial perfusion line; 2 is pulsatile tube; 3 is aortic cannula; 4 is venous line; 5 is pressures lines; 6 is partial tube clamp (simulated resistance). (b) Mock circulations with 3 different tube positions. {I} Pulsatile tube was positioned at 6 cm distance from aortic cannula; {II} Pulsatile tube positioned at 150 cm distance from aortic cannula; {III} Pulsatile tube wedged between roller pump and oxygenator. 1 is roller pump; 2 is oxygenator; 3 is arterial line; 4 is pulsatile tube; 5 is aortic cannula; 6 is resistance (tube clamp); 7 is venous line. P1, P2, P3, P4, and P5 are perfusion pressures recording spots.

*Mock Circulation.* With slight modification, according to Ündar et al. and Wang et al. [33, 34], the circuit was composed of (Figure 2(a)) a roller head pump (Cobe Cardiovascular Inc.), pediatric oxygenator (Sorin Lilliput 2 Ecmo), and hemofilter (Sorin Group hemoconcentrators), primed with fresh piglet's blood mixed with dextran in concentration of 2/3 to 1/3, respectively. A pediatric arterial line circuit, PVC tube (1.5 m length), 14 FR aortic cannula (DLP Medtronic, Inc.), venous line (1.5 m length), and simulating vascular resistance partial clamp were positioned downstream to the aortic cannula.

*Methods.* Five pressure lines were connected to a pressure monitor (BIOPAC physiology monitoring system) and positioned at remote spots on the circuit as follows: pre/post-oxygenator (P1,P2); pre/post-tube (P3,P4), and post-simulated resistance (P5), which represents the arterial perfusion curve in patients (Figure 2(b)).

Flow-pressure curves were recorded first in a steady mode, then pulsatile after switching the tube's generator on, using variant pump flow rate (400, 600, 800, and 1000 mL/min) and fixed pulsatile frequency (110 bpm) in group P.

Momentum energy losses were roughly calculated according to differences of flow pressures between the spots (P1–P5) and compared with tube position in 3 different mock circuits as follows.

- Circuit I: the tube was positioned downstream to the oxygenator at 6 cm distance from the aortic cannula.
- Circuit II: the tube was positioned downstream to the oxygenator at 150 cm distance from the aortic cannula.
- Circuit III: the tube was positioned upstream between the roller pump and oxygenator.

### 2.2. *In Vivo* Study

This study was approved by the Animal Research Facility at Sun Yat-sen University and conformed to the Guide for the Care and Use of Laboratory Animals (NIH Publication no. 85-23, revised in 1996).

*Ten* domestic piglets of both sexes were randomly designated to either pulsatile group (P, *n* = 5; 11.750.60 kg) or the nonpulsatile group (NP, *n* = 5; 11.800.84 kg). Animals were premedicated and maintained on general anesthesia according to our previously published protocols [35]. After a median cervicotomy and tracheotomy, a 3.5–5 tracheal tube was inserted, followed by mechanical ventilation (PA-500 PuLang Technologies Inc) with 40% oxygen, 10–15 mL/kg/min of tidal volume, and frequency of 15/min. The right carotid artery was isolated and cannulated with a 6 Fr. arterial sheath. Then a Millar probe (4 Fr. MIKRO-TIP catheter transducer, Millar Instruments) was introduced through the carotid line into the aorta for continuous systemic pressure (AP) monitoring (BIOPAC physiology monitoring system); this enabled other hemodynamic measurements mentioned below. After median sternotomy, mediastinal dissection, pericardiotomy, and dissection of great vessels were followed by positions of purse-string (5/0 polypropylene) at the RA appendage, infundibulum, ascending aorta, and LV apex.

*Cardiac Catheterizations/Hemodynamic Monitoring.* A (5 Fr.) double-lumen central venous line (Hydrocath, B-D Tech.) was introduced through the RA purse-string for drug administration and RA pressure monitoring. An intrapulmonary catheter (5 Fr. Swan-Ganz) was introduced through the infundibular purse-string for pulmonary artery pressure (PAP). Left atrium (LA) pressure was obtained by direct needle puncture at predetermined time points. Cardiac output (CO) was measured with a TRANSONIC transit-time flowmeter temporarily positioned around the aortic root at predetermined time points.

Total time (T) of the experiment was 3 h, divided into (T1, T2, and T3) in correspondence to data collection: T1 = baseline; T2 = nearly 1 h of myocardial ischemia, just before severe hemodynamic deterioration; and T3 = by the end of experiment after 2 h of treatment.

*Induction of Acute Myocardial Ischemia.* After surgical preparation, and data collection for T1, the left anterior descending coronary artery (LAD) was encircled with a 4/0 *polypropylene stitch*, distal to the 2nd diagonal bifurcation and tightly obstructed with a snugger for 2 h period.

During the first 1 h of ischemia, ventricular fibrillation (VF) and cardiac arrest were frequent after 20–30 min of ischemia. Animals were assisted with classical cardiopulmonary resuscitation (CPR) and DC shocks (20–30 J), without any further IHD pharmacological supports.

After 30 min, heparin was given (250 UI/kg) intravenously, followed by LVAD installation as follows.

*In P group*, a modified aortic cannula (12 Fr., 10 cm length; DLP-Medtronic, Inc.) was inserted at the aortic root and a short piece of PVC tube (14 Fr., 15 cm length) was introduced into the LV apex. Both aortic cannula and LV vent were shortly cut in a matter to avoid energy losses caused by unnecessary length. A pulsatile tube prototype was connected at its distal end to a conventional roller pump (Cobe Cardiovascular Inc.) and to the aortic cannula at its proximal end. The LV vent was connected to the other end of the roller pump.

*In NP group*, the aorta was cannulated using a standard aortic cannula (12 Fr. DLP-Medtronic, Inc.) and an apical LV vent (14 Fr. DLP-Medtronic, Inc.). Both aortic and LV vent were connected to a centrifugal pump (Sorin group Revolution).

The LVAD circuit in both P and NP groups was primed with heparinized saline, then deaired, clamped, and kept on standby until T2. Animals that did not survive T2 were excluded. 

*Hemodynamics *data were collected from both groups at T1, T2, and T3 including AP, PAP, LA, and RA pressures, heart rate, and CO. The vascular resistance index was calculated with the flowing formula:

- pulmonary vascular resistance index (PVRI) = 80 ∗ (MPAP – PCWP) / CO ∗ Wt,
- systemic vascular resistance index (SVRI) = 80 ∗ (MAP – CVP) / CO ∗ Wt,

where MPAP is the mean pulmonary arterial pressure, PCWP is the pulmonary capillary wedge pressure, substituted for LA pressure, MAP is the mean arterial pressure, CVP is the central venous pressure, substituted for RA pressure, CO is the cardiac output, and Wt is the body weight.

*Therapeutic Phase.* During the second hour of ischemia in P group, the pulsatile tube generator was switched on at a fixed frequency (90 bpm) and irrespective of heart rate (93.755.25 bpm). The flow rate of the roller pump was kept around 0.3 L/min (150 mL/Kg). In the NP group, the centrifugal pump flow rate was fixed around 0.8 L/min.

After 1 h of LV mechanical assist, the LAD snugger was released, allowing coronary reperfusion during the second hour of assistance (total 2 h of LVAD).

The ischemic zone was measured and evaluated macroscopically until the end of the experiment (T3).

Animals were euthanized with a 10 mL injection of saturated potassium chloride (KCl) upon completion of 3 h of LVAD or just before severe hemodynamic deterioration.

*Statistics.* Continuous variables are expressed as the mean SEM. Comparisons between groups of independent samples were performed with students *t*-test hemodynamic data *in vitro* and a 2-way ANOVA for the *in vivo* data. *P* with a value less than 0.05 was considered statistically significant. GraphPad Prism software was applied for all the statistical analyses in this study.

## 3. Results

**Figure 3:**
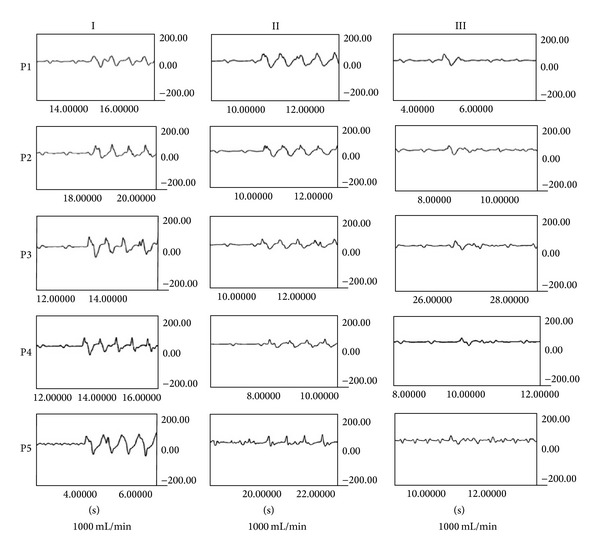
Perfusions curves (*in vitro*). Perfusion curves (in mmHg) obtained at different circuit sites in 3 different pulsatile tube positions: I, II, and III close and distant from aortic cannula and pre-oxygenator, respectively. The perfusion curve amplitude was significantly higher at P5 with position I, compared to positions II and III.

**Figure 4:**
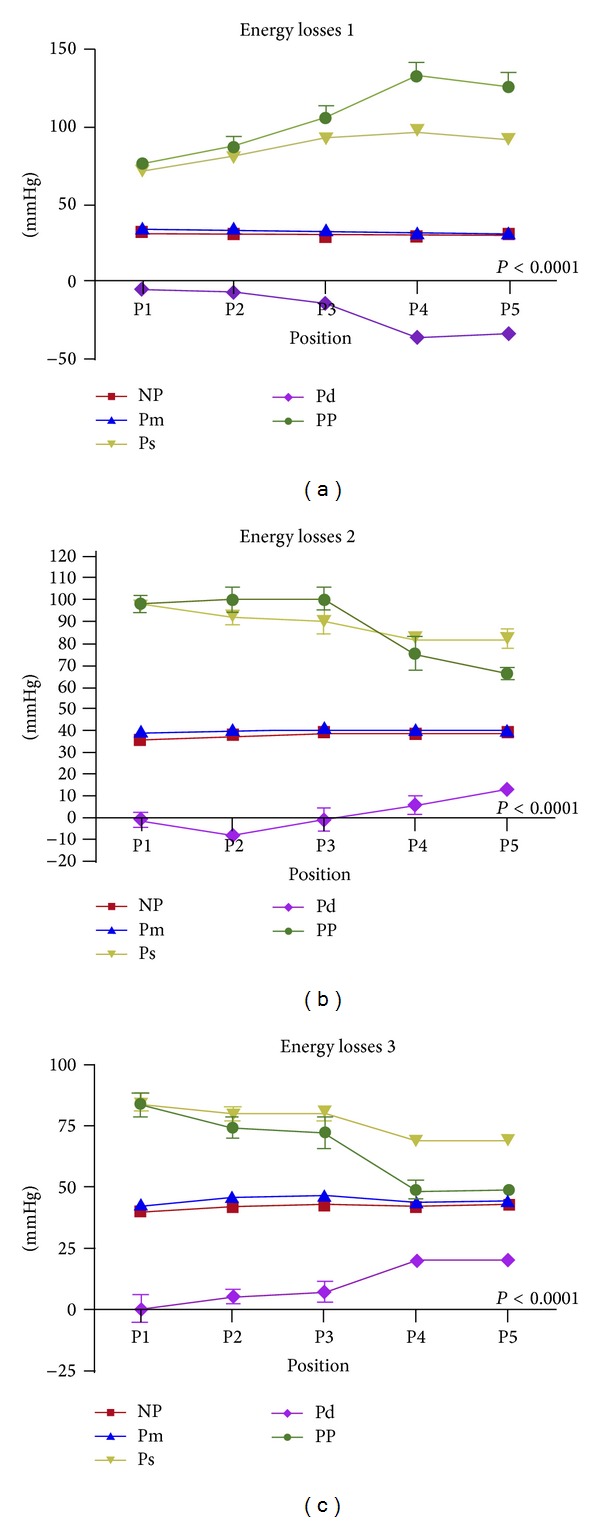
Comparative steady and pulsatile flow perfusion curves obtained from 3 different circuits. Energy losses 1 (upper panel) are pulsatile tube at 6 cm from aortic cannula; energy losses 2 are pulsatile tube at 150 cm from aortic cannula; energy losses 3 are pre-oxygenator pulsatile tube position. P1–P5 are distant circuit spots for perfusion pressure records (mmHg). NP is nonpulsatile; Pm is mean pulsatile pressure, Ps is systolic pressure; Pd is diastolic pressure; PP is pulse pressure. The pulse pressure (green color) was significantly higher with position I compared to positions II and III.

**Figure 5:**
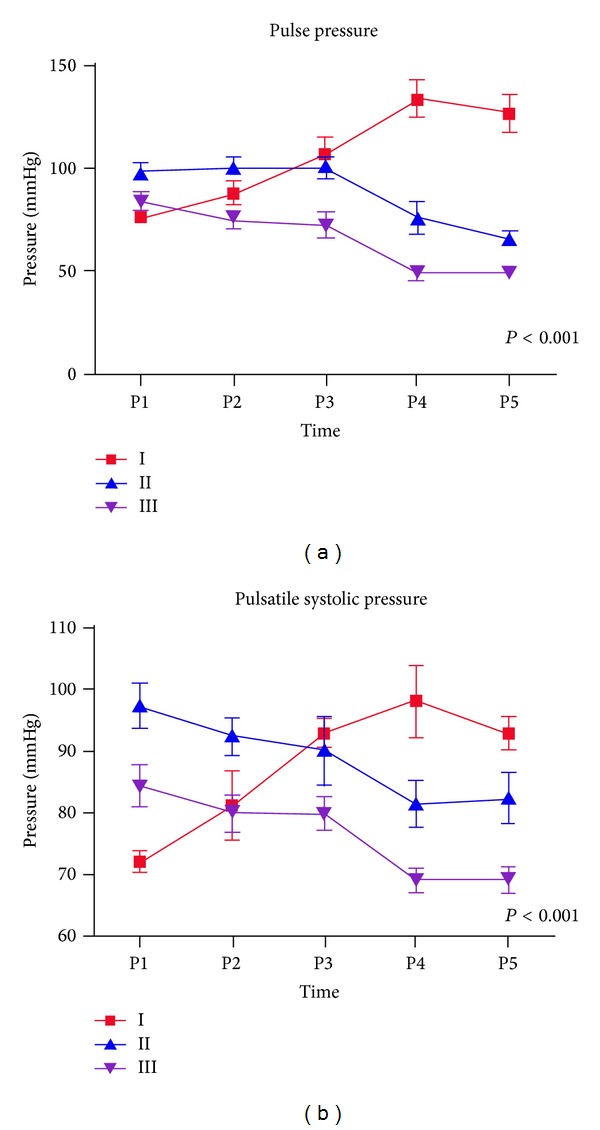
Pulsatile flow pulse pressure (a) and systolic pressure (b). Energy losses with different tube positions: I is 6 cm from aortic cannula; II is 150 cm from aortic cannula; III is pre-oxygenator. P1–P5 are perfusion pressure records (mmHg) at main circuit energy losses spots. At P5 the pulse pressure (a) as well as the systolic pressure (b) were significantly higher in position I (red color) compared to other position II (blue color) and III (violet color). Circuit I: P1, P2 are pre/post-oxygenator pressure; P3, P4 are pre/post-tube. Circuit II: P1 is pre-oxygenator; P2, P3 are pre/post-tube; P4 is pre-aortic cannula. Circuit III: P1, P2 are pre/post-tube; P3 is post-oxygenator; P4 is pre-aortic cannula. P5 is post simulated resistance in all circuits. Pm is mean pulsatile pressure; Ps: systolic pulsatile pressure, Pd: pulsatile diastolic pressure; PP: pulse pressure. Pulse pressure was higher at P5 in circuit I, compared with II and III. Pm was higher at P5 compared to NP with position I (*P* < 0.001).

*In vitro*, as resumed in Table 1 and Figures 3, 4, and 5, globally, mean flow pressures at P5 were significantly better in the pulsatile mode (P) compared with steady flow (NP); mean P5 were 31,8 ± 1 versus 30,8 ± 0,5; 40,3 ± 0,5 versus 39,3 ± 0,5; and 44,8 ± 0,5 versus 43 ± 0,00 mmHg with tube position in circuits I, II, and III, respectively (*P* < 0.001).

Reduction of the pulsatile perfusion curve amplitude was directly proportional to distance between aortic cannula and tube position; that is, I > II > III.

The collected pulse pressure data from each circuit were as follows.

Circuit I = P4 (133,3 ± 17,7) − P5 (126,3 ± 18,6) *≈* 7 mmHg.

Circuit II = P3 (100,3 ± 10,3) − P5 (66 ± 6,1) *≈* 44 mmHg.

Circuit III = P2 (74,5 ± 8,7) − P5 (48,8 ± 4,7) *≈* 26 mmHg. In circuit III, there were significant increased flow pressures in a retrograde manner; that is, P1 > P2 and P5 > P1 (*P* < 0.001).

**Figure 6:**
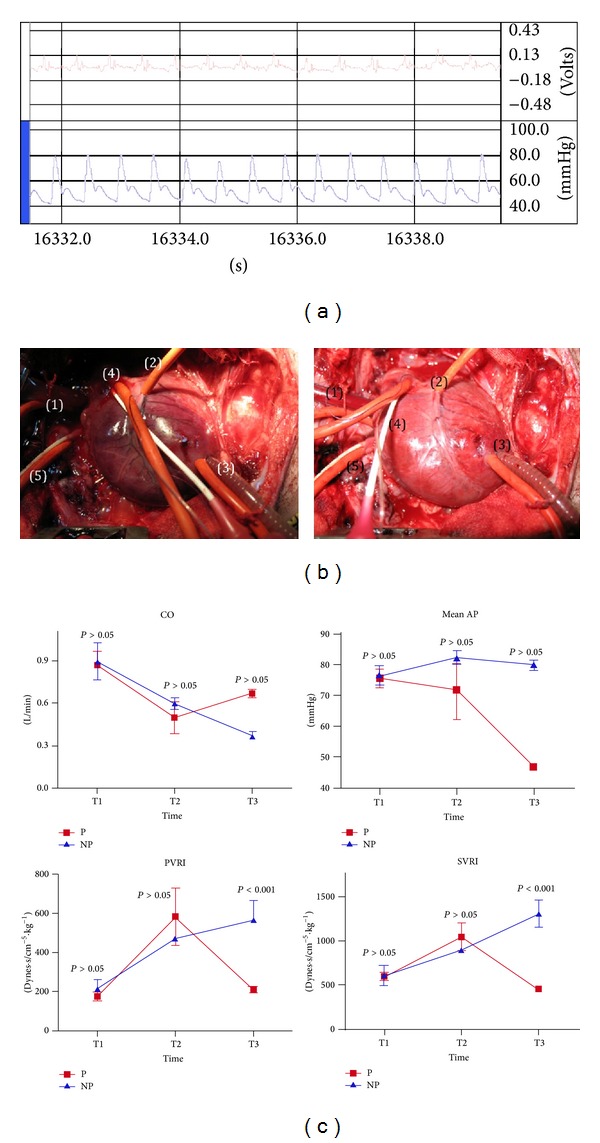
(a) Pulsatile LVAD perfusion curve in ischemic piglets. Unsynchronized pulsations with heartbeat perfusion curve with nearly physiological pulse pressure. (b) Pulsatile LVAD in piglet ischemic model. Left panel: massive myocardial ischemic zone after LAD ligation; right panel: clearance of the ischemic zone after 15 min of pulsatile LVAD (roller pump + pulsatile tube). 1 is aortic cannula; 2 is LAD permanent snugger; 3 is left ventricular apical vent; 4 is intrainfudibular pulmonary artery and Millar right ventricular pressure catheters; 5 is right atrium pressure line. 6 (c) Hemodynamic results of pulsatile and nonpulsatile left ventricular assist device in ischemic piglet model. P: pulsatile group; NP: nonpulsatile group; MAP: mean arterial pressure (mmHg); CO: cardiac output (L/min); SVRI: systemic vascular resistance index (dynes·s·cm^−5^/kg^−1^); PVRI: pulmonary vascular resistance index (dynes·s·cm^−5^/kg). T1: baseline; T2: 1 h of myocardial ischemia; T3: 1 h after treatment.

*In vivo*, as resumed in (Table 2), apparently, the induced pulsatile perfusion curve was nearly physiological in amplitude as shown in (Figure 6(a)). Clearance of the ischemic zone with better hemodynamic was observed in group P compared with the NP group (Figure 3(b)). In P group heart rate was 76.255.12 versus 99.25 ± 3.77 in NP (bpm; *P* < 0.05). Hemodynamic (Figure 3(c)) was better in the P group compared with NP. Cardiac output (CO) was nonsignificantly increased in the P group compared with NP; CO was 0.67 0.26 versus 0.380.03 (L/min; *P* > 0.05), respectively. There was significant vasodilatation in the P group compared with NP; mean AP in the P group was 46.830.52 versus 79.881.65 in NP group (mmHg; *P* > 0.05); mean PAP in P was 24.832.88 versus 34.537.68 in NP group (mmHg; *P* > 0.05); SVRI was 451.7224.01 versus 1309.88 ± 151.93; and PVRI was 210.6616.02 versus 566.9897.98 (dynes·s·cm^−5^/kg^−1^; *P* < 0.001), respectively.

For further details please refer to the supplementary attached operative movies available online at http://dx.doi.org/10.1155/2013/651769.

## 4. Discussion

In this study, conventional steady-flows of a simulated CPB circuit and LVAD were transformed successfully into a pulsatile mode of perfusion, using a double lumens tube device.

*In vitro*, there were significant pressure losses with tube position in circuits III and II compared with I. *In vivo*, the induced pulsatile perfusion curve was efficient to improve hemodynamic and restore endothelial function in the P group compared with NP group.

Normally, fluid movement in hydraulic circuits, which means momentum transfers with frictional losses, depends on driving forces, resistances, viscosity, and conduits geometries [36].

Nevertheless, quantifications of lumped models that could be achieved with accuracy according to the Bernoulli's principles of energy losses *in vitro* remain controversial due to different cardiovascular criteria [37, 38].

Endothelial shear stress (ESS) that control vascular resistances could be influenced by hemorheological changes, particularly, pulse pressure and/or shear rate [39, 40].

For example, under normal hemorheological conditions, microcirculation behavior approaches that of Newton's second law, such as seen in athletics; that is, a high physical performance (ESS) can be induced by increasing the pulse pressure and slowing the heart rate (shear rate). In contrast, in any abnormal hemorheological state, microcirculation presents behavior that approaches that of Bernoulli's 3rd equation, which is interpreted by the Fahraeus-Lindqvist effect [24], in which plasma becomes stuck at the inner vascular boundary layers, whereas erythrocytes move faster at the center [35]. These may explain clubbing fingers phenomena in cyanotic heart disease.

**Figure 7:**
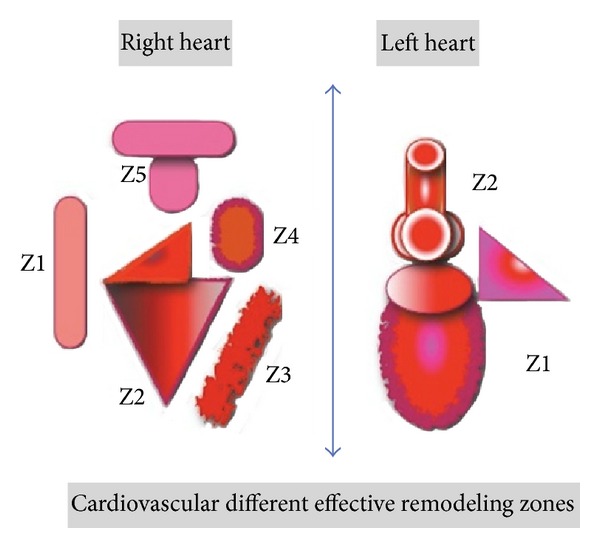
Different remodeling zones (Z) of left and right hearts circuits [15]. Left heart: Z1 = left ventricle pump; Z2 = peristaltic aortic pump + valsalva. Right heart: Z1= systemic veins; Z2 = atrio-ventricular cavity; Z3 = interventricular septum; Z4 = infundibulum; Z5 = pulmonary artery.

Therefore, delivery of ESS with pulsatile CAD should be induced according to the biophysics and pathophysiological conditions of each heart circuit (Figure 7).

Physiologically, the left heart side has specific morphological particularities that must be considered.

The left ventricle (LV) and peristaltic arteries represent the main circulatory driving forces at the left heart circuit, which deliver almost constant shear rate and pulse pressure. Flow dynamics inside the valsalva sinuses determines coronary ostia morphogenesis [41] and may contribute to a severe hemodynamic deterioration [42]. This means delivery of ESS with CAD must be induced at the left heart side according to Newton's law by maintaining adequate pulse pressure.

The main target at the left heart side is maintenance organs microcirculation with adequate ESS. And this may explain common phenomena like postoperative or postpartum depression syndrome [43, 44], by disturbances of hemorheological factors due to sudden drop of cerebral perfusion pressure, in addition to ESS losses in the first example.

Contrarily to the left heart side, the right heart side can adjust blood volume and shear rates at five different anatomical zones according to its physiological demands [45]. The PA represents a low-level remodeling zone, similar to systemic veins. At the same time, PA compliance is much greater than that of the large veins [46]. Therefore, direct induction of shear stress according to Newton's law, using intravenous (IV) or intrapulmonary pulsatile CAD, must be avoided at the right heart side. Most importantly, delivery of ESS should not disturb the physiological remodeling of the right heart circuit because increased ESS with high pulse pressure promotes serious hemodynamic conditions and irreversible remodeling, such as Eisenmenger syndrome [47]. In addition, the RV is preload dependent that could not tolerate to be unloaded [48].

These may explain insufficiency of current CAD employments in congestive heart (CHF) patients with severe RV failure that still exhibit a high mortality rate (65%–95%) [49]. In pediatrics, applications of CAD remain controversial, as most of devices were designed for management cardiovascular diseases (CVD), in adults, and then miniaturized to cope with pediatric populations. However, pediatric patients are more vulnerable to hemodynamic disturbances caused by right heart failure due to congenital anomalies versus adults CVD most commonly caused by left ventricular ischemia and atherosclerosis [50].

The proposed pulsatile tube device adapts to biophysics and pathophysiological conditions of the *left heart*. This promotes pulsatile tube applications with conventional CPB and LVAD or as biventricular-assist device in association with a pulsatile suit device for a right ventricular-assist device [48].

**Figure 8:**
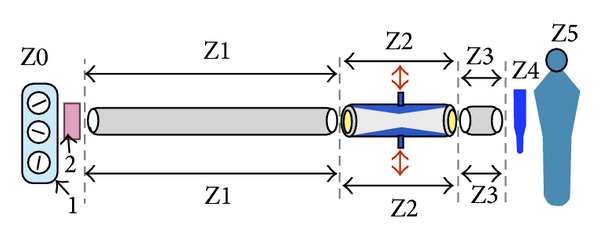
Main momentum energy losses zones within a CPB perfusion circuit [15]. Z0 = conventional CBP (1); Z1 = post-oxygenator (2) arterial line; Z2 = pulsatile tube; Z3 = pre-aortic cannula zone; Z4 = aortic cannula; Z5 = patient.

Practically, and for better understanding of the pulsatile tube's function as a cardiovascular perfusion device, we propose a state of momentum energy losses that could be identified in 6 main zones (Figure 8) as follows: pre/post-oxygenator (Z0, Z1), tube zone (Z2), post-tube *or* pre-aortic cannula (Z3), aortic cannula (Z4), and cardiovascular system (Z5).

Normally, pulsatile tube (Z2) receives steady-flow from Z1, then the propagated pulsatile impacts move the stagnant blood boundaries' layers at the inner flexible tube and push them towards the center according to the “Bernoulli” principles. These would promote fewer traumatic effects of blood components compared with current devices. In addition, the downstream position of the pulsatile tube, which avoids already two obstructive zones of energy losses, makes high cost accessories unnecessary [25, 51]

The present study results showed the importance of energy losses in correlation to circuits length. Particularly, the distance between the pulsatile tube and the pre-aortic cannula (Z3), was represented by P4 in position (I), P3 and P2 in positions II and III, respectively. Eventually, these constitutions of vortices in Z3 are the consequences of strong pulsatile impacts inside rigid tubes with fixed geometries. Thus, compromising this Z3 distance between patients and CAD is crucial.

In contrast, energy losses were very important with tube position in circuit III, which simulates current pulsatile CPB and/or CAD. We have observed severe leakage after few minutes of pulsations (≤5 min) with circuit III. The increased pressure at P5 due to this retrograde turbulent flow was enhanced by the application of a nonocclusive pump (Table 1). In addition, the pulsatile tube became an obstructive zone per se in positions II and III.

Energy losses inside the aortic cannula (Z4) are usually caused by unnecessary length and tapering end. We have attempted to diminish these drawbacks of unnecessary length by shortening the aortic cannula in the P group.

At the cardiovascular side (Z5), turbulent flow of energy losses that starts from the tip of aortic cannula (divergent diffuser) will be absorbed by the arterial wall filter to be dependent on ESS that control organs microcirculations [52].

According to the present study results, the induced pulsatile tube pulse pressure of the (Figure 6(a)) improved hemodynamic with almost complete clearance of the ischemic zone after few minutes of pulsatile flow assistance in the group P compared with NP.

Contrary to the NP group, the induced vasodilatation of the pulsatile group was most probably caused by endothelial vasodilators secretion (e.g. NO), which means maintenance of ESS with tube pulsations. By the end of the experiment (T3), heart rate was 76.255.12 bpm in group P compared with 99.25 ± 3.77 bpm in the group NP, which signifies better hemodynamics with less myocardial oxygen consumption.

Interestingly, myocardial recoveries in the pulsatile group occurred in pediatric immature myocardium of animal model with poor coronary collaterals [53, 54]. This was confirmed in another experimental model using intrapulmonary shear stress enhancement after permanent LAD ligation in piglets [55]. These prove the important role of endocardium ESS interdependency to improve subendocardial microcirculation and hemodynamic regardless of classical coronary network conditions.

We should emphasize that in all our *in vivo* studies, the tube pulsations were not synchronized with heart rate. This confirms our therapeutic policy based on similar observations with other pulsatile devices (e.g., suit and catheter), proving that pulsatile CAD should not be synchronized with the heartbeat in case of heart failure [15].

Interestingly, the pulsatile tube was tested as a stimulator of the LV subendocardium without association roller pump, in two ischemic piglets (please refer to the supplementary attached movies). There was rapid clearance of the ischemic zone and improved contractility, after few minutes of unsynchronized pulsations (110 bpm; ≤5 min), followed by severe vasodilatation.

This proved the hypersensitivity of the LV endocardium and heterogeneity of the left heart endothelium that may be an emerging concept regarding the endothelial impact of current devices [56, 57].

### 4.1. Study Limitations

Although these results proved that pulsatile tube was efficient to maintain ESS, some weak points must be improved. This includes microporosity of the inner tube (PTFE), which became almost collapsed by the external latex membrane cover. Accordingly, tube association with a hypersensitive sophisticated centrifugal pump was impossible. There were centrifugal pump failures in two excluded piglets from the NP group due to uncontrolled systemic hypertensive crises (≥140 mmHg). Therefore, the use of a nonocclusive roller pump increased the circuits' artifacts, particularly III and II, and disturbed the applications of more recent formulae for energy losses evaluations [33, 34]. In addition, latex did not stop impregnations of the inner tube with blood that made homogenous histopathological and biological data very doubtful.

### 4.2. Improvements

The proposed study concept deserves further evaluations with more enlarged investigations. To overcome the current limits, we are developing a new device with an inner tube made of ChronoFelx AR, which is similar to PTFE hardness (65 A–75 A) but without micropores. These enabled us to continue our ongoing studies: (a) pulsatile versus nonpulsatile LVAD in ischemic piglet models; (b) biventricular assist device combining the pulsatile tube as a LVAD and the pulsatile trouser as right ventricular assist device (RVAD) [48]. We are planning a new animal model for evaluating the effectiveness pulsatile tube, associated with conventional CPB during the first trimester of pregnancy.

## 5. Conclusion

Pulsatile tube adaptable to conventional perfusion devices could induce homogenous, downstream, and nearly physiologic pulsatile perfusion flow, with lower energy losses. This represents a cost-effective promising method with low mortality and morbidity, especially in fragile cardiac patients.

## Supplementary Material

1st Movie "Pulsatile Tube tested in vitro and in vivo": first part of the movie (in vitro study) shows the pulsatile tube integrated the arterial perfusion line of a mock cardiopulmonary bypass circuit (Circuit I). The middle part of the movie shows the arterial pressure curve obtained by the tube connected as LVAD on an arrested heart. End of the movie shows the tube connected to conventional roller pump as LVAD.
2nd Movie: shows a control group piglet assisted with a conventional LVAD using a continuous flow centrifugal pump. The dark infracted zone was present after ≥ 1 h of assistance.
3rd Movie: shows the pulsatile tube tested as CAD per se (without to be connected to a perfusion pump). There were macroscopic clearance of the infarcted zone and improvement of myocardial contractility, to be followed by severe vasodilation occurred after 5 minutes of pulsations, most probably due to massive release of endothelial mediators.

## Figures and Tables

**Table 1:** Pulsatile and nonpulsatile (NP) flows pressures records (mmHg) of three different mock circuits (I, II, and III).

Groups	P1	P2	P3	P4	P5
(1)					
NP (I)	32.5 ± 1.3	31.3 ± 1.3	30.3 ± 0.5	30.3 ± 0.5	30.8 ± 0.5
NP (II)	36.3 ± 1.3	37.8 ± 1	39.5 ± 0.6	38.5 ± 0.6	39.3 ± 0.5
NP (III)	40.3 ± 1	42.3 ± 1	43 ± 0.00	42.3 ± 0.6	43 ± 0.00
(2)					
Pm. (I)	34.5 ± 1.7	34.5 ± 1.3	33.5 ± 1.7	32.3 ± 1	31.8 ± 1
Pm. (II)	39.3 ± 0.5	40 ± 0.8	40.8 ± 0.6	40.5 ± 0.6	40.3 ± 0.5
Pm. (III)	43 ± 1.2	46 ± 2.2	46.3 ± 1.5	44 ± 0.8	44.8 ± 0.5
(3)					
Ps. (I)	72 ± 3.5	81 ± 11	92.8 ± 4.9	98 ± 11.5	92.8 ± 5.6
Ps. (II)	97.3 ± 7	92.3 ± 6	90 ± 11.2	81.3 ± 7.5	82.3 ± 8.4
Ps. (III)	84.3 ± 6.6	79.8 ± 5.9	79.8 ± 5.5	69 ± 3.9	69 ± 4.2
(4)					
Pd. (I)	−4.4 ± 3.2	−6.5 ± 7.4	−13.6 ± 11.7	−35.3 ± 8	−33.5 ± 13.3
Pd. (II)	−1.1 ± 6.2	−7.8 ± 4.4	−0.8 ± 9.4	5.6 ± 8.4	13 ± 3.2
Pd. (III)	0.5 ± 10	5.3 ± 5.3	7.5 ± 7.5	20.3 ± 3.8	20.3 ± 0.5
(5)					
PP (I)	76.4 ± 3.4	87.5 ± 11.8	106.4 ± 15.9	133.3 ± 17.7	126.3 ± 18.6
PP (II)	98.3 ± 7.9	100 ± 10.4	100.3 ± 10.3	75.7 ± 15.4	66 ± 6.1
PP (III)	83.8 ± 9.2	74.5 ± 8.7	72.3 ± 12.6	48.8 ± 7.1	48.8 ± 4.7

Circuit I: P1, P2: pre/post-oxygenator pressure; P3, P4: pre/post-tube. Circuit II: P1: pre-oxygenator; P2, P3: pre/post-tube; P4: pre-aortic cannula. Circuit III: P1, P2: pre/post-tube; P3: post-oxygenator; P4: pre-aortic cannula. P5: post simulated resistance in all circuits. Pm: mean pulsatile pressure; Ps: systolic pulsatile pressure, Pd: pulsatile diastolic pressure; PP: pulse pressure. Pulse pressure was higher at P5 in circuit I, compared with II and III. Pm was higher at P5 compared to NP with position I. (*P* < 0.001).

**Table 2:** Hemodynamic results of pulsatile and non-pulsatile left ventricular assist device in ischemic piglet model.

Groups	T1		T2		T3	
	P	NP	P	NP	P	NP
Wt*	11.75 ± 0.60^‡^	11.80 ± 0.84	nd	nd	nd	nd
HR*	85.4 ± 7.92^‡^	89.00 ± 9	93.75 ± 5.25^‡^	102.33 ± 6.43	76.25 ± 5.12^‡^	99.25 ± 3.77
MAP*	75.66 ± 3.1^‡^	76.6 ± 3	71.73 ± 9.3^‡^	82.35 ± 2.16	46.83 ± 0.52	79.88 ± 1.65^‡^
MPAP*	24.93 ± 5.6^‡^	27.44 ± 7.17	38.13 ± 5.44^‡^	41.47 ± 1.46	24.83 ± 2.88	34.53 ± 7.68^‡^
LAP*	2.62 ± 0.61^‡^	2.28 ± 1.1	2.28 ± 0.69^‡^	2.33 ± 0.57	2.6 ± 0.48	1.78 ± 0.26^§^
RAP*	2.60 ± 0.55^‡^	3.78 ± 1.09	4.8 ± 2.05^‡^	5.4 ± 2.03	2.6 ± 1.67^‡^	3.85 ± 0.86
CO*	0.87 ± 0.1^‡^	0.9 ± 0.13	0.5 ± 0.11^‡^	0.6 ± 0.04	0.67 ± 0.26	0.38 ± 0.03^‡^
SVRI^†^	600.22 ± 45.27^‡^	610.6 ± 112.37	1046.57 ± 156.05^‡^	899.28 ± 15.82	451.72 ± 24.01	1309.88 ± 151.93^§^
PVRI^†^	174.93 ± 20.95^‡^	215.71 ± 42.93	583.02 ± 144.93^‡^	471.38 ± 12.58	210.66 ± 16.02	566.98 ± 97.98^§^

P: pulsatile group; NP: nonpulsatile group; Wt: weight (kg); HR: heart rate (bpm); MAP: mean arterial pressure (mmHg); MPAP: mean pulmonary arterial pressure (mmHg); LAP: left atrial pressure (mmHg); RAP: right atrial pressure (mmHg); CO: cardiac output (L/min); SVRI: systemic vascular resistance index (dynes·s·cm^−5^/kg^−1^); PVRI: pulmonary vascular resistance index (dynes·s·cm^−5^/kg); *measured variables; ^†^calculated variables; ^‡^ *P* > 0.05 between the P and NP groups; ^§^ *P* < 0.05 between the P and NP groups. T1: baseline; T2: 1 h of myocardial ischemia; T3: 1 h after treatment. Group P: pulsatile tube frequency: 90 bpm; roller pump flow 0.34 ± 0.02 L/min. Group NP: centrifugal pump flow = 0.80 L/min.

## References

[1] Diehl P, Aleker M, Helbing T (2010). Enhanced microparticles in ventricular assist device patients predict platelet, leukocyte and endothelial cell activation. *Interactive CardioVasc Thoracic Surgery*.

[2] Pieske B (2004). Reverse remodeling in heart failure—fact or fiction?. *European Heart Journal, Supplement*.

[3] Habazettl H, Kukucka M, Weng YG (2006). Arteriolar blood flow pulsatility in a patient before and after implantation of an axial flow pump. *Annals of Thoracic Surgery*.

[4] McHugh J, Cheek DJ (1998). Nitric oxide and regulation of vascular tone: pharmacological and physiological considerations. *The American Journal of Critical Care*.

[5] Thorin E, Nguyen T-D, Bouthillier A (1998). Control of vascular tone by endogenous endothelin-1 in human pial arteries. *Stroke*.

[6] Hoeks APG, Samijo SK, Brands PJ, Reneman RS (1995). Noninvasive determination of shear-rate distribution across the arterial lumen. *Hypertension*.

[7] Petrovic D, Zorc-Pleskovic R, Zorc M (2000). Apoptosis and proliferation of cardiomyocytes in heart failure of different etiologies. *Cardiovascular Pathology*.

[8] Burkhoff D, Cohen H, Brunckhorst C (2006). A randomized multicenter clinical study to evaluate the safety and efficacy of the TandemHeart percutaneous ventricular assist device versus conventional therapy with intraaortic balloon pumping for treatment of cardiogenic shock. *The American Heart Journal*.

[9] Bonetti PO, Barsness GW, Keelan PC (2003). Enhanced external counterpulsation improves endothelial function in patients with symptomatic coronary artery disease. *Journal of the American College of Cardiology*.

[10] Thunberg CA, Gaitan BD, Arabia FA, Cole DJ, Grigore AM (2010). Ventricular assist devices today and tomorrow. *Journal of Cardiothoracic and Vascular Anesthesia*.

[11] Wilmot I, Morales DLS, Price JF (2011). Effectiveness of mechanical circulatory support in children with acute fulminant and persistent myocarditis. *Journal of Cardiac Failure*.

[12] Yuruk K, Bezemer R, Euser M (2012). The effects of conventional extracorporeal circulation versus miniaturized extracorporeal circulation on microcirculation during cardiopulmonary bypass-assisted coronary artery bypass graft surgery. *Interactive CardioVasc Thoracic Surgery*.

[13] Roselli RJ, Brophy SP (2003). Redesigning a biomechanics course using challenge-based instruction. *IEEE Engineering in Medicine and Biology Magazine*.

[14] Olufsen MS, Nadim A (2004). On deriving lumped models for blood flow and pressure in the systemic arteries. *Mathematical Biosciences and Engineering*.

[15] Nour S, Misra AN (2012). Flow and rate: concept and clinical applications of a new hemodynamic theory. *Biophysics*.

[16] Roussel JC, Sénage T, Baron O (2009). CardioWest (Jarvik) total artificial heart: a single-center experience with 42 patients. *Annals of Thoracic Surgery*.

[17] Park SJ, Tector A, Piccioni W (2005). Left ventricular assist devices as destination therapy: a new look at survival. *Journal of Thoracic and Cardiovascular Surgery*.

[18] Cooper JR, Abrams J, Frazier OH (2006). Fatal pulmonary microthrombi during surgical therapy for end-stage heart failure: possible association with antifibrinolytic therapy. *Journal of Thoracic and Cardiovascular Surgery*.

[19] Pouard P, Mauriat P, Ek F (2006). Normothermic cardiopulmonary bypass and myocardial cardioplegic protection for neonatal arterial switch operation. *European Journal of Cardio-Thoracic Surgery*.

[20] Ündar A, Masai T, Yang S-Q, Goddard-Finegold J, Frazier OH, Fraser CD (1999). Effects of perfusion mode on regional and global organ blood flow in a neonatal piglet model. *Annals of Thoracic Surgery*.

[21] Ishida K, Imamaki M, Ishida A, Shimura H, Miyazaki M (2004). Heparin-induced thrombocytopenia after coronary artery bypass grafting with cardiopulmonary bypass: report of a case. *Surgery Today*.

[22] Box FMA, van der Geest RJ, Rutten MCM, Reiber JHC (2005). The influence of flow, vessel diameter, and non-Newtonian blood viscosity on the wall shear stress in a carotid bifurcation model for unsteady flow. *Investigative Radiology*.

[23] Rastan AJ, Walther T, Alam NA (2008). Moderate versus deep hypothermia for the arterial switch operation—experience with 100 consecutive patients. *European Journal of Cardio-Thoracic Surgery*.

[24] Neri Serneri GG (1981). Pathophysiological aspects of platelet aggregation in relation to blood flow rheology in microcirculation. *Ricerca in Clinica e in Laboratorio*.

[25] Lim CH, Yang S, Choi J-W, Sun K (2009). Optimizing the circuit of a pulsatile extracorporeal life support system in terms of energy equivalent pressure and surplus hemodynamic energy. *Artificial Organs*.

[26] Onorati F, Presta P, Fuiano G (2007). A Randomized trial of pulsatile perfusion using an intra-aortic balloon pump versus nonpulsatile perfusion on short-term changes in kidney function during cardiopulmonary bypass during myocardial reperfusion. *The American Journal of Kidney Diseases*.

[27] Kadoi Y, Saito S (2000). Perfusion during cardiopulmonary bypass does not improve brain oxygenation. *The Journal of Thoracic and Cardiovascular Surgery*.

[28] Sanfelippo PM, Baker NH, Ewy HG (1987). Vascular complications associated with the use of intraaortic balloon pumping. *Texas Heart Institute Journal*.

[29] Voss B, Krane M, Jung C (2010). Cardiopulmonary bypass with physiological flow and pressure curves: pulse is unnecessary!. *European Journal of Cardio-Thoracic Surgery*.

[30] Shroyer AL, Grover FL, Hattler B (2009). On-pump versus off-pump coronary-artery bypass surgery. *The New England Journal of Medicine*.

[31] Anderson RM (1993). *The Gross Physiology of the Cardiovascular System*.

[32] Gravlee GP, Davis RF, Stammers AH, Ungerleider RM (2007). *Cardiopulmonary Bypass: Principles and Practices*.

[33] Ündar A, Ji B, Lukic B (2006). Quantification of perfusion modes in terms of surplus hemodynamic energy levels in a simulated pediatric CPB model. *ASAIO Journal*.

[34] Wang S, Haines N, Ündar A (2009). Quantification of pressure-flow waveforms and selection of components for the pulsatile extracorporeal circuit. *Journal of Extra-Corporeal Technology*.

[35] Nour S, Dai G, Carbognani D (2012). Intrapulmonary shear stress enhancement: a new therapeutic approach in pulmonary arterial hypertension. *Pediatric Cardiology*.

[36] Kessler DP, Greenkorn RA (1999). *Momentum, Heat, and Mass Transfer Fundamentals*.

[37] Dougherty FC, Donovan FM, Townsley MI (2003). Harmonic analysis of perfusion pumps. *Journal of Biomechanical Engineering*.

[38] Rider AR, Schreiner RS, Ündar A (2007). Pulsatile perfusion during cardiopulmonary bypass procedures in neonates, infants, and small children. *ASAIO Journal*.

[39] Furchgott RF, Zawadzki JV (1980). The obligatory role of endothelial cells in the relaxation of arterial smooth muscle by acetylcholine. *Nature*.

[40] Samet MM, Lelkes PI, Lelkes PI, Samet MM (1999). The hemodynamic environment of endothelium in vivo and its simulation in vitro. *Regulation of Endothelial Cells by Mechanical Forces*.

[41] Hutchins GM, Kessler-Hanna A, Moore GW (1988). Development of the coronary arteries in the embryonic human heart. *Circulation*.

[42] Palmieri V, Bella JN, Arnett DK (2001). Aortic root dilatation at sinuses of Valsalva and aortic regurgitation in hypertensive and normotensive subjects: the hypertension genetic epidemiology network study. *Hypertension*.

[43] Doraiswamy PM, MacFall J, Krishnan KRR (1999). Magnetic resonance assessment of cerebral perfusion in depressed cardiac patients: preliminary findings. *The American Journal of Psychiatry*.

[44] Guille C, Newman R, Fryml LD, Lifton CK, Epperson CN (2013). Management of postpartum depression. *Journal of Midwifery & Women's Health*.

[45] Nour S, Wu G, Zhensheng Z, Chachques JC, Carpentier A, Payen D (2009). Review paper: the forgotten driving forces in right heart failure: new concept and device. *Asian Cardiovascular and Thoracic Annals*.

[46] Fourie PR, Coetzee AR, Bolliger CT (1992). Pulmonary artery compliance: its role in right ventricular-arterial coupling. *Cardiovascular Research*.

[47] D’Alto M, Vizza CD, Romeo E (2007). Long term effects of bosentan treatment in adult patients with pulmonary arterial hypertension related to congenital heart disease (Eisenmenger physiology): safety, tolerability, clinical, and haemodynamic effect. *Heart*.

[48] Nour S, Dai G, Wang Q, Wang F, Chachques JC, Wu GF (2012). Forgotten driving forces in right heart failure—part II: experimental study. *Asian Cardiovascular and Thoracic Annals*.

[49] Prutkin JM, Strote JA, Stout KK (2008). Percutaneous right ventricular assist device as support for cardiogenic shock due to right ventricular infarction. *Journal of Invasive Cardiology*.

[50] Potapov EV, Stiller B, Hetzer R (2007). Ventricular assist devices in children: current achievements and future perspectives. *Pediatric Transplantation*.

[51] Ündar A, Ji B, Lukic B (2006). Comparison of hollow-fiber membrane oxygenators with different perfusion modes during normothermic and hypothermic CPB in a simulated neonatal model. *Perfusion*.

[52] Popel AS, Johnson PC (2005). Microcirculation and hemorheology. *Annual Review of Fluid Mechanics*.

[53] Allen BS (2004). Pediatric myocardial protection: where do we stand?. *Journal of Thoracic and Cardiovascular Surgery*.

[54] Gorge G, Schmidt T, Ito BR, Pantely GA, Schaper W (1989). Microvascular and collateral adaptation in swine hearts following progressive coronary artery stenosis. *Basic Research in Cardiology*.

[55] Nour S, Yang D, Dai G (2013). Intrapulmonary shear stress enhancement: a new therapeutic approach in acute myocardial ischemia. *International Journal of Cardiology*.

[56] Cheng JM, den Uil CA, Hoeks SE (2009). Percutaneous left ventricular assist devices vs. intra-aortic balloon pump counterpulsation for treatment of cardiogenic shock: a meta-analysis of controlled trials. *European Heart Journal*.

[57] Klotz S, Jan Danser AH, Burkhoff D (2008). Impact of left ventricular assist device (LVAD) support on the cardiac reverse remodeling process. *Progress in Biophysics and Molecular Biology*.

